# Site-specific patterns of early-stage cancer diagnosis during the COVID-19 pandemic

**DOI:** 10.1093/jncics/pkae022

**Published:** 2024-03-23

**Authors:** Connor J Kinslow, David M DeStephano, Alfred I Neugut, Kekoa Taparra, David P Horowitz, James B Yu, Simon K Cheng

**Affiliations:** Department of Radiation Oncology, Columbia University Vagelos College of Physicians and Surgeons and NewYork-Presbyterian, New York, NY, USA; Herbert Irving Comprehensive Cancer Center, Columbia University Vagelos College of Physicians and Surgeons and NewYork-Presbyterian, New York, NY, USA; Department of Radiation Oncology, Columbia University Vagelos College of Physicians and Surgeons and NewYork-Presbyterian, New York, NY, USA; Herbert Irving Comprehensive Cancer Center, Columbia University Vagelos College of Physicians and Surgeons and NewYork-Presbyterian, New York, NY, USA; Herbert Irving Comprehensive Cancer Center, Columbia University Vagelos College of Physicians and Surgeons and NewYork-Presbyterian, New York, NY, USA; Department of Medicine, Columbia University Vagelos College of Physicians and Surgeons and NewYork-Presbyterian, and Department of Epidemiology, Mailman School of Public Health, Columbia University, New York, NY, USA; Department of Radiation Oncology, Stanford University, Stanford, CA, USA; Department of Radiation Oncology, Columbia University Vagelos College of Physicians and Surgeons and NewYork-Presbyterian, New York, NY, USA; Herbert Irving Comprehensive Cancer Center, Columbia University Vagelos College of Physicians and Surgeons and NewYork-Presbyterian, New York, NY, USA; Department of Radiation Oncology, Columbia University Vagelos College of Physicians and Surgeons and NewYork-Presbyterian, New York, NY, USA; Herbert Irving Comprehensive Cancer Center, Columbia University Vagelos College of Physicians and Surgeons and NewYork-Presbyterian, New York, NY, USA; Department of Radiation Oncology, Columbia University Vagelos College of Physicians and Surgeons and NewYork-Presbyterian, New York, NY, USA; Herbert Irving Comprehensive Cancer Center, Columbia University Vagelos College of Physicians and Surgeons and NewYork-Presbyterian, New York, NY, USA; Department of Radiation Oncology, James J. Peters Department of Veterans Affairs Medical Center, Bronx, NY, USA

## Abstract

The COVID-19 pandemic caused widespread disruptions in cancer care. We hypothesized that the greatest disruptions in diagnosis occurred in screen-detected cancers. We identified patients (≥18 years of age) with newly diagnosed cancer from 2019 to 2020 in the US National Cancer Database and calculated the change in proportion of early-stage to late-stage cancers using a weighted linear regression. Disruptions in early-stage diagnosis were greater than in late-stage diagnosis (17% vs 12.5%). Melanoma demonstrated the greatest relative decrease in early-stage vs late-stage diagnosis (22.9% vs 9.2%), whereas the decrease was similar for pancreatic cancer. Compared with breast cancer, cervical, melanoma, prostate, colorectal, and lung cancers showed the greatest disruptions in early-stage diagnosis. Uninsured patients experienced greater disruptions than privately insured patients. Disruptions in cancer diagnosis in 2020 had a larger impact on early-stage disease, particularly screen-detected cancers. Our study supports emerging evidence that primary care visits may play a critical role in early melanoma detection.

The COVID-19 pandemic caused widespread disruptions in cancer care, including reductions and delays in screenings, diagnosis, billing, and treatment (1-5). Intentional delays in primary care visits and screenings were recommended by authoritative bodies, such as the American Society of Clinical Oncology and American College of Surgeons (ACS) (2,3). These delays are expected to cause an increased burden of cancer-related mortality in the coming years (6). In the National Cancer Database (NCDB), the number of recorded cancer diagnoses decreased during the first year of the pandemic by 12.4% to 14.4%, with early-stage diagnoses preferentially affected (2,3). Diagnoses by individual cancer sites did not consistently reflect overall trends, however, suggesting site-specific variations related to patterns of care (3). Previous studies analyzed gross categories rather than disease-specific sites, precluding insights into the consequences of disruptions in clinical practice, screening, and early detection. We hypothesized that during the COVID-19 pandemic, the greatest disruptions occurred in screen-detected cancers, including cancers of the breast, prostate, colon/rectum, lung, and cervix.

The NCDB (2022 submission) is a retrospective, nationwide dataset sponsored by the ACS and the American Cancer Society, constituting 70% of incident invasive cancers in the United States (7-10). We identified patients (≥18 years of age) with newly diagnosed cancer from 20 sites from January 2019 to December 2020. Patients with unknown stage/occult disease, without diagnostic confirmation, or unflagged reference date were excluded. The change in proportion of early-stage (American Joint Committee on Cancer 0-II) to late-stage (III-IV) cancers from 2019 to 2020 was compared using a weighted linear regression. Covariables included cancer site, age, sex, race, and insurance status. Race was abstracted from medical records, which have a high sensitivity and positive predictive value for self-reported race (11,12). Native Hawaiian and Pacific Islander participants were disaggregated from Asian participants in accordance with federal standards defined in 1997 and mandated by the Patient Protection and Affordable Care Act for US Health and Human Services–related and National Institutes of Health–related work in 2010. To aid in understanding the holistic changes that occurred in the United States, we created a graph that shows the relative changes in early- and late-stage disease and in the number of patients affected. Statistical analyses were conducted using RStudio, version 1.4.1106, software (RStudio, Inc, Boston, MA). This study was exempt from review by the Columbia University Institutional Review Board and follows the Strengthening the Reporting of Observational Studies in Epidemiology guideline for observational studies.

After excluding 115 952 patients without diagnostic confirmation (6% in 2019; 6.3% in 2020) and 124 475 with unknown disease stage (6.8% in 2019; 7.2% in 2020), we identified 899 661 and 759 205 new primary cancers diagnosed in 2019 and 2020, respectively (Table 1). The greatest disruptions occurred in melanoma (‒21.1%) and prostate cancer (‒21.1%) and the lowest in pancreatic cancer (‒7.3%). Across all cancer sites, disruptions in early-stage diagnosis were greater than in late-stage diagnosis (17% vs 12.5%) (Figure 1, A). Melanoma demonstrated the greatest relative decrease in early-stage vs late-stage diagnosis (22.9% vs 9.2%), whereas the decrease was similar for pancreatic cancer (7.6% vs 7.0%) (Figure 1, B). Compared with breast cancer, cervical cancer (β = ‒1.7, 95% confidence interval [CI] = ‒3.2 to ‒0.26); *P* = .02), melanoma (β = ‒1.6, 95% CI = ‒2.3 to ‒0.90; *P* < .001), prostate cancer (β = ‒1.6, 95% CI = ‒2.3 to ‒0.98; *P* < .001), colorectal cancer (β = ‒1.5, 95% CI = ‒2.1 to ‒0.91; *P* < .001), and lung cancer (β = ‒0.71, 95% CI = 1.2 to ‒0.18; *P* = .01) showed the greatest disruptions in early-stage diagnosis. Similar results were observed when using pancreatic cancer as the reference disease (Supplementary Table 1, available online). Breast cancer, however, did not demonstrate a statistically significant shift in early-stage to late-stage diagnosis over pancreatic cancer.

**Figure 1.**
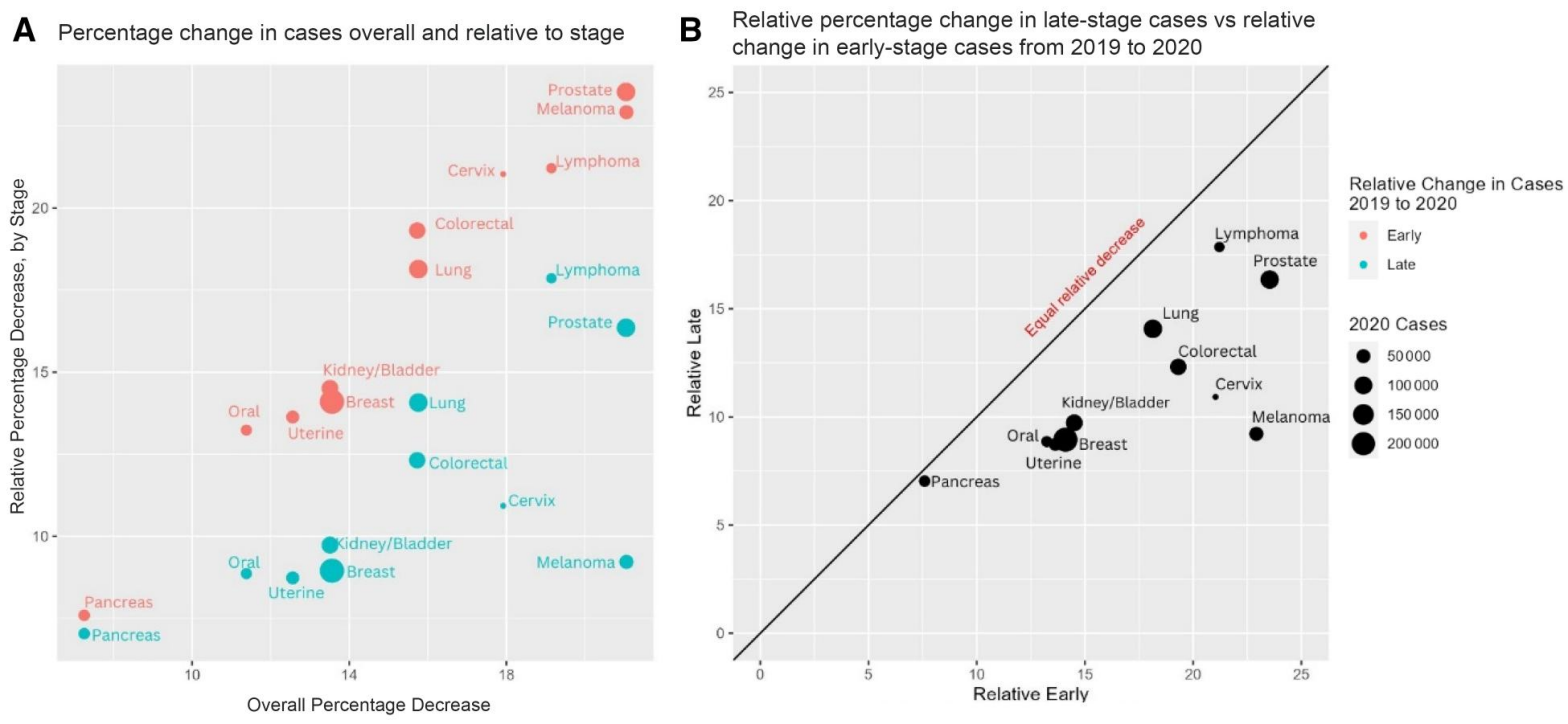
Disruptions in early-stage and late-stage cancer diagnosis. Disruptions in early-stage and late-stage diagnosis as a function of overall decrease (**A**) and relative decrease in late-stage compared with early-stage diagnosis (**B**). The black line indicates where disruptions of early-stage and late-stage diagnoses are the same.

**Table 1.** Disruptions in cancer diagnosis in 2020

New cancer diagnoses^a^								
Variable	2019, No.	2020, No.	Overall, Δ%	Early stage, Δ%	Late stage, Δ%	Proportional Δ (early stage)^b^	β (95% confidence interval)^c^	*P*
**Age, y**								
15-39	30 431	26 019	14.5	16.0	10.8	‒1.2	0.03 (‒0.92 to 0.99)	.94
40-49	69 419	59 231	14.7	15.9	11.0	‒1.1	[Referent]	[Referent]
50-59	168 121	138 464	17.6	19.1	14.3	‒1.2	‒0.29 (‒0.92 to 0.34)	.37
60-69	287 633	242 431	15.7	17.0	13.0	‒.01	0.23 (‒0.39 to 0.84)	.47
≥70	344 057	293 060	14.8	16.3	11.8	‒1.2	‒0.03 (‒0.68 to 0.61)	.92
**Sex**								
Male	399 995	332 308	16.9	19.2	13.3	‒1.7	[Referent]	[Referent]
Female	499 666	426 897	14.6	15.6	11.6	‒0.8	‒0.17 (‒0.59 to 0.25)	.43
**Race**								
American Indian, Alaska Native	3115	2825	9.3	14.5	‒0.3	‒3.7	‒1.5 (‒4.0 to 0.92)	.22
Asian	19 701	16 044	18.6	20.6	14.1	‒1.8	‒0.91 (‒1.9 to 0.12)	.08
Black	99 895	84 245	15.7	16.9	13.4	‒0.9	0 (‒0.47 to 0.47)	.99
Native Hawaiian, Pacific Islander	1990	1830	8.0	8.2	7.8	‒0.1	‒1.6 (‒4.6 to 1.4)	.30
White	746 971	628 775	15.8	17.2	12.8	‒1.1	[Referent]	[Referent]
Other	27 989	25 486	8.9	11.4	3.4	‒1.8	‒0.53 (‒1.4 to 0.29)	.20
**Insurance status**								
Private insurance	340 629	283 467	16.8	17.8	14.0	‒0.9	[Referent]	[Referent]
Medicaid/Medicare	531 716	454 765	14.5	15.9	11.6	‒1.2	‒0.19 (‒0.57 to 0.18)	.32
Uninsured	17 289	14 115	18.4	23.8	12.1	‒3.6	‒1.9 (‒3.0 to ‒0.75)	**.001** ^d^
Unknown	10 027	6858	31.6	34.4	26.0	‒2.7	‒0.61 (‒2.2 to 0.97)	.45
**Cancer site**								
Breast	251 308	217 237	13.6	14.1	8.9	‒0.6	[Referent]	[Referent]
Prostate	134 468	106 167	21.1	23.5	16.4	‒2.1	‒1.6 (‒2.3 to ‒0.98)	**<.001**
Lung^e^	128 530	108 275	15.8	18.1	14.1	‒1.2	‒0.71 (‒1.2 to ‒0.18)	**.01**
Kidney/bladder^f^	95 407	82 518	13.5	14.5	9.7	‒1	‒0.53 (‒1.1 to 0.08)	.09
Colorectal^g^	90 101	75, 927	15.7	19.3	12.3	‒2.1	‒1.5 (‒2.1 to ‒0.91)	**<.001**
Melanoma	63 087	49 802	21.1	22.9	9.2	‒2	‒1.6 (‒2.3 to ‒0.90)	**<.001**
Uterine	44 564	38 967	12.6	13.6	8.7	‒1	‒0.3 (‒1.0 to 0.41)	.41
Head and neck^h^	28 093	24 896	11.4	13.2	8.9	‒1.2	‒0.7 (‒1.6 to 0.21)	.13
Pancreas	29 131	27 019	7.3	7.6	7.0	‒0.1	0.35 (‒0.50 to 1.2)	.42
Lymphoma^i^	25 044	20 248	19.2	21.2	17.9	‒1	‒0.39 (‒1.4 to 0.58)	.43
Cervix	9928	8149	17.9	21.0	10.9	‒2.6	‒1.7 (‒3.2 to ‒0.26)	**.02**
Total	899 661	759 205	15.6	17.0	12.5	—	—	—

a Newly diagnosed cancers are derived from the National Cancer Database, 2019-2020, for 20 cancer sites. Cancer sites were combined by relevant clinical disease sites.

b The proportional change (Δ) was calculated as ([early diagnoses in 2020/total diagnoses in 2020] ‒ [early diagnoses in 2019/total diagnoses in 2019]).

c β is the change in the proportion of early-stage diagnoses between 2019 and 2020 relative to the reference group, weighted by group sample size in 2020 and adjusted for all other variables.

d Bold indicates statistically significant values.

e Small cell or non–small cell lung cancer.

f Kidney or bladder cancer.

g Colorectal cancer.

h Lip, gum of mouth, floor of mouth, tongue, pharynx, tonsil, or salivary gland.

i Hodgkin or non-Hodgkin lymphoma.

Disruptions in early-stage diagnosis did not differ by age, sex, or race. Uninsured patients experienced greater disruptions than did privately insured patients (β = ‒1.9, 95% CI = ‒3.0 to ‒0.75; *P* = .001). Native Hawaiian and Pacific Islander patients showed numerically the greatest disruption in diagnosis, though the difference was not statistically significant (β = ‒1.6, 95% CI = ‒4.6 to 1.4; *P* = .30). Diagnoses stratified by sex are included in the supplement (Supplementary Table 2, available online).

Disruptions in cancer diagnosis in 2020 had a larger impact on early-stage disease, particularly screen-detected cancers. As a consequence, increased cancer-related mortality is anticipated in the coming years (6). Uninsured patients may be disproportionately affected. Although melanoma is not typically considered a screen-detected cancer, suspicious skin lesions are identified during primary care and dermatology visits. The US Preventative Services Task Force states that there is currently insufficient evidence to access the benefits and harms of routine skin screening (13). Our study supports emerging evidence that primary care visits may play a critical role in early melanoma detection (14).

Other investigators have recently shown similar findings comparing stage I vs II or IV disease (15). Our figure allows the visualization of cancer presentation during COVID-19, identifying melanoma as a particular outlier. We additionally used broader inclusion criteria and disaggregated Native Hawaiian and Pacific Islander patients from Asian patients. Though not statistically significant, Native Hawaiian and Pacific Islander patients showed numerically the greatest disruptions in early-stage diagnosis, which is masked when incorrectly and inappropriately aggregating this racial group with Asian patients (16).

Strengths of this study include use of one of the largest and highest-quality cancer registries in the world, one that was validated during the early months of the pandemic (2). The NCDB is not population based. Therefore, our findings may not be generalizable to the 30% of the US population not captured in this study (17-22). Other secular changes in cancer screening not related to COVID-19 may have affected our observations.

## Supplementary Material

pkae022_Supplementary_Data

## Data Availability

Data are available upon direct request to the ACS.
