# Supplementary material for: Site-specific patterns of early-stage cancer diagnosis during the COVID-19 pandemic
Source: JNCI Cancer Spectr. 2024 Mar 23;8(3):pkae022. doi: 10.1093/jncics/pkae022 (PMC11062758; doi:10.1093/jncics/pkae022)
Supplement: pkae022_Supplementary_Data [file pkae022_supplementary_data.docx]

**Supplementary Material**

Supplementary Table 1- Disruptions in Cancer Diagnosis in 2020

| New Cancer Diagnoses^a^ | | | | | | | | |
| --- | --- | --- | --- | --- | --- | --- | --- | --- |
| Cancer Site | 2019 (n) | 2020 (n) | Overall Δ% | Early Stage Δ% | Late Stage Δ% | Proportional Δ (Early Stage)^g^ | β (95% CI)^h^ | P-value |
| Pancreas | 29,131 | 27,019 | 7.3 | 7.6 | 7.0 | -0.1 | Reference | Reference |
| Breast | 251,308 | 217,237 | 13.6 | 14.1 | 8.9 | -0.6 | -0.35 (-1.2, 0.50) | 0.42 |
| Prostate | 134,468 | 106,167 | 21.1 | 23.5 | 16.4 | -2.1 | -2.0 (-2.9, -1.1) | **<0.001** |
| Lung^c^ | 128,530 | 108,275 | 15.8 | 18.1 | 14.1 | -1.2 | -1.1 (-1.9, -0.19) | **0.02** |
| Kidney/Bladder^e^ | 95,407 | 82,518 | 13.5 | 14.5 | 9.7 | -1 | -0.88 (-1.8, 0.02) | 0.06 |
| Colorectal^d^ | 90,101 | 75, 927 | 15.7 | 19.3 | 12.3 | -2.1 | -1.8 (-2.7, -0.93) | **<0.001** |
| Melanoma | 63,087 | 49,802 | 21.1 | 22.9 | 9.2 | -2 | -1.9 (-2.9, -0.97) | **<0.001** |
| Uterine | 44,564 | 38,967 | 12.6 | 13.6 | 8.7 | -1 | -0.65 (-1.7, 0.39) | 0.22 |
| Head and Neck^f^ | 28,093 | 24,896 | 11.4 | 13.2 | 8.9 | -1.2 | -1.0 (-2.2, 0.08) | 0.07 |
| Lymphoma^b^ | 25,044 | 20,248 | 19.2 | 21.2 | 17.9 | -1 | -0.75 (-1.9, 0.44) | 0.22 |
| Cervix | 9,928 | 8,149 | 17.9 | 21.0 | 10.9 | -2.6 | -2.1 (-3.7, -0.42) | **0.01** |
| Total | 899,661 | 759,205 | 15.6 | 17.0 | 12.5 |  |  |  |

^a^Newly diagnosed cancers are derived from the National Cancer Database, 2019-2020 for 20 cancer sites. Cancer sites were combined by relevant clinical disease sites: ^b^Hodgkin/Non-Hodgkin lymphoma; ^c^Small cell/Non-small cell lung cancer; ^d^Colon/rectum cancer; ^e^Kidney/bladder cancer; ^f^Lip/gum of mouth/floor of mouth/tongue/pharynx/tonsil/salivary gland

^g^The proportional change (Δ) was calculated as ([early diagnoses in 2020/total diagnoses in 2020] – [early diagnoses in 2019/total diagnoses in 2019]).

^h^β is the change in the proportion of early-stage diagnoses between 2019 and 2020, relative to the reference group, weighted by group sample size in 2020 and adjusted for all other variables.

Supplementary Table 2- Cancer Diagnoses from 2019-2020, Stratified by Sex

| New Cancer Diagnoses^a^ | | | | | | | | |
| --- | --- | --- | --- | --- | --- | --- | --- | --- |
| Cancer Site | Sex | 2019 (n) | 2020 (n) | Late Stage 2019 (n) | Late Stage 2020 (n) | Early Stage 2019 (n) | Early Stage 2020 (n) | Overall Δ% |
| Breast | Female | 249,218 | 215,480 | 26,111 | 23,792 | 223,107 | 191,688 | 13.54 |
| Breast | Male | 2,090 | 1,757 | 337 | 289 | 1,753 | 1,468 | 15.93 |
| Prostate | Male | 134,468 | 106,167 | 46,565 | 38,951 | 87,903 | 67,216 | 21.05 |
| Lung^c^ | Female | 65,456 | 54,854 | 36,013 | 30,796 | 29,443 | 24,058 | 16.2 |
| Lung^c^ | Male | 63,074 | 53,421 | 39,173 | 33,810 | 23,901 | 19,611 | 15.3 |
| Kidney/Bladder^e^ | Female | 28,223 | 23,637 | 6,081 | 5,427 | 22,142 | 18,210 | 16.25 |
| Kidney/Bladder^e^ | Male | 67,184 | 58,881 | 13,808 | 12,527 | 53,376 | 46,354 | 12.36 |
| Colorectal^d^ | Female | 42,502 | 35,917 | 21,337 | 18,754 | 21,165 | 17,163 | 15.49 |
| Colorectal^d^ | Male | 47,599 | 40,010 | 24,780 | 21,683 | 22,819 | 18,327 | 15.94 |
| Melanoma | Female | 26,268 | 20,633 | 3,119 | 2,804 | 23,149 | 17,829 | 21.45 |
| Melanoma | Male | 36,819 | 29,169 | 5,439 | 4,965 | 31,380 | 24,204 | 20.78 |
| Uterine | Female | 44,564 | 38,967 | 9,757 | 8,905 | 34,807 | 30,062 | 12.56 |
| Pancreas | Female | 13,893 | 12,793 | 8,241 | 7,686 | 5,652 | 5,107 | 7.92 |
| Pancreas | Male | 15,238 | 14,226 | 9,588 | 8,889 | 5,650 | 5,337 | 6.64 |
| Head and Neck^f^ | Female | 8,353 | 7,449 | 3,819 | 3,556 | 4,534 | 3,893 | 10.82 |
| Head and Neck^f^ | Male | 19,740 | 17,447 | 8,096 | 7,303 | 11,644 | 10,144 | 11.62 |
| Lymphoma^b^ | Female | 11,261 | 9,018 | 6,670 | 5,416 | 4,591 | 3,602 | 19.92 |
| Lymphoma^b^ | Male | 13,783 | 11,230 | 8,714 | 7,221 | 5,069 | 4,009 | 18.52 |
| Cervix | Female | 9,928 | 8,149 | 3,057 | 2,723 | 6,871 | 5,426 | 17.92 |

^a^Newly diagnosed cancers are derived from the National Cancer Database, 2019-2020 for 20 cancer sites. Cancer sites were combined by relevant clinical disease sites: ^b^Hodgkin/Non-Hodgkin lymphoma; ^c^Small cell/Non-small cell lung cancer; ^d^Colon/rectum cancer; ^e^Kidney/bladder cancer; ^f^Lip/gum of mouth/floor of mouth/tongue/pharynx/tonsil/salivary gland
